# Harnessing mRNA technology against *Fasciola hepatica*: Immunological insights from a fatty acid binding protein vaccine

**DOI:** 10.3389/fimmu.2025.1693674

**Published:** 2025-11-25

**Authors:** Javier Sánchez-Montejo, Cristina Teodosio, Tania Strilets, Julio López-Abán, Raúl Manzano-Román, Julio Pozo, Silvia Martin, Lidia Silos, Ignacio Trujillo, Mariano A. García-Blanco, Belén Vicente, Antonio Muro

**Affiliations:** 1 Infectious and Tropical Diseases Research Group (e-INTRO), Biomedical Research Institute of Salamanca, Research Centre for Tropical Diseases at the University of Salamanca Instituto de Investigación Biomédica de Salamanca (IBSAL) - Centro de Investigación de Enfermedades Tropicales de la Universidad de Salamanca (CIETUS), Salamanca, Spain; 2 Cancer Research Center Instituto Universitario de Biología Molecular y Celular del Cancer, Universidad de Salamanca - Consejo Superior de Investigaciones Científicas (IBMCC, USAL-CSIC), Salamanca, Spain; 3 Department of Medicine, University of Salamanca (USAL), Salamanca, Spain; 4 Cytometry Service (NUCLEUS), University of Salamanca (USAL), Salamanca, Spain; 5 Centro de Investigación Biomédica en Red Cáncer (CIBERONC; CB16/12/00400), Madrid, Spain; 6 Biomedical Research Institute of Salamanca (IBSAL), Salamanca, Spain; 7 Department of Microbiology, Immunology and Cancer Biology, University of Virginia, Charlottesville, VA, United States; 8 Cytognos SL, A BD Biosciences Company, Salamanca, Spain; 9 Center for RNA Science and Medicine, University of Virginia, Charlottesville, VA, United States

**Keywords:** *Fasciola hepatica*, parasite, trematode, mRNA vaccine, cytometry, FABP

## Abstract

mRNA platforms offer a promising strategy to overcome limitations in helminth vaccinology. We used the helminth *Fasciola hepatica*, which is a major veterinary and zoonotic pathogen for which no licensed vaccine exists, as a test case. We engineered a codon-optimized mRNA encoding the *F. hepatica* fatty-acid–binding protein (FABP), verified its expression in HEK293T cells, and formulated it in SM-102 lipid nanoparticles (LNPs). BALB/c mice received a prime-boost immunization (3 weeks apart) followed by longitudinal blood and terminal spleen immune profiling by spectral flow cytometry. Immunization induced rapid innate immune activation with marked neutrophil expansion and monocyte maturation, while reducing circulating mature NK cells, consistent with recruitment to lymphoid tissues. Adaptive responses included increased circulating CD8^+^ T cells dominated by EMRA effectors, expansion of TCRαβ+ double-negative T cells with memory/effector phenotypes, and a reduction in peripherally induced CD25- regulatory T cells. CD4^+^ T-helper cells showed a shift toward memory/effector subsets, and antigen-specific Th1 and Th2 responses in the spleen were detected only in vaccinated mice. B-cell analysis revealed accelerated maturation of B2 cells with expansion of marginal-zone, follicular, and germinal-center compartments, higher frequencies of class-switched (IgM-) plasma cells, and exclusive detection of anti-FABP IgG in the mRNA-LNP group. These results demonstrate that an mRNA-LNP vaccine encoding *F. hepatica* FABPs elicits innate immune activation, cytotoxic and helper T-cell immunity, and class-switched humoral responses in mice, supporting its potential as a candidate for *F. hepatica* vaccination in a future challenge experiment against the infection.

## 1 Introduction

*Fasciola hepatica*, commonly known as the liver fluke, is a widespread endoparasite that infects a variety of wild and domestic animals. It has a significant impact on the health and welfare of grazing sheep, cattle, and goats, leading to substantial losses in milk, meat production, and fertility. The global economic losses are estimated at $ 3 billion (1). Additionally, the treatment of infected animals and the implementation of integrated control programs incur considerable costs (2). Furthermore, the impact of *Fasciola hepatica* extends beyond livestock, posing a significant zoonotic threat. The disease affects 2.4 to 17 million people across more than 70 countries and has been recognized as a neglected foodborne trematode by the WHO (3). This dual impact on both animal agriculture and human health underscores the critical need for advanced and sustainable control strategies.

Since vaccination against fasciolosis is not available, current control measures rely on anthelmintic treatment; however, resistance to fasciolicides, such as triclabendazole or clorsulon, has emerged in some areas (4). Preventing infection through vaccination would be an ideal addition to integrated control methods, helping to reduce parasite loads in herds and potentially circumventing the development of resistance. *Fasciola hepatica* has evolved mechanisms that skew the host immune response towards non-protective Th2 and regulatory Treg profiles while suppressing Th1 and cytotoxic immunity (5). In both rodent models and natural ruminant hosts, infection initially triggers a mixed Th1/Th2 response (with increases in IFN-γ, IL-4, IL-10, and TGF-β). Eventually, a dominant Th2/Treg environment quickly develops, characterized by high levels of IL-4 and IL-10, alternative macrophage activation (6, 7), and the expansion of IL-10-producing Tregs (8). At the same time, pro-inflammatory Th1 cytokines (e.g., IFN-γ) and cytotoxic CD8+ T cell responses are suppressed, resulting in weak IgG1/IgG2a antibody production and ineffective cell-mediated killing, which enables the parasite to establish chronic infections (9).

Over the past few decades, several antigens have been investigated as vaccine candidates, among which glutathione S-transferases (GST), cathepsin L proteases (CL), leucine aminopeptidases (LAP), and fatty-acid-binding proteins (FABP) stand out as leading targets (10–14). FABPs are molecules synthesized by the parasite to sequester essential fatty acids from the host since trematodes cannot synthesize long-chain lipids (15). These proteins have already been used as vaccination candidates through various technologies, providing partial protection against *Fasciola hepatica* (10, 16, 17) or *Schistosoma mansoni* (18). Among these, recombinant Fh15, a *Fasciola hepatica* FABP, has shown significant potential, inducing partial protective immunity in mice and sheep models (19). Furthermore, Fh15 has been shown to modulate the host’s immune response by reducing inflammation and suppressing lipopolysaccharide (LPS)-induced cytokine storm (20). Despite these promising findings and the significant efforts in classical vaccine development, no vaccine has yet achieved complete protection or been approved for commercial use; therefore, fasciolosis remains without an effective immunoprophylactic solution. In recent years, mRNA technology has emerged as a promising strategy for vaccination that could be effective against parasites (21). Early studies on helminths have shown that it can be an effective method to elicit immunity using parasite proteins (22). Thus, this technology may be developed to express *Fasciola hepatica* proteins and elicit an immune response against the parasite. Our study aims to evaluate the immunogenic potential of the FABP Fh15 produced by an optimized mRNA construct and formulated in lipidic nanoparticles for *in vivo* testing.

## 2 Materials and methods

### 2.1 Animal handling

Animal procedures were conducted under Spanish (RD 53/2013) and European Union (Directive 2010/63/CE) regulations regarding animal experimentation. The accredited Animal Experimentation Facilities (Registration number: PAE/SA/001) of the University of Salamanca (USAL) were used for such procedures. Animals were maintained in standard polycarbonate and wire cages, with controlled 12-hour light and dark periods, a temperature of 23-25°C, and food and water available *ad libitum*. The USAL’s Research Ethics Committee approved the procedures used in this study (Ref. CEI 1057). Every effort was made to minimize animal suffering.

### 2.2 mRNA design and cloning

The sequence of the FABP Fh15 was retrieved from UniProt (Q7M4G0), reverse-translated, and codon-optimized using the GenSmart Codon Optimization tool for human and mouse expression hosts. Optimized sequences were synthesized using GenTitan Gene fragment and cloned into our previously described mRNA expression vector (23) by restriction cloning. Briefly, this vector was designed by placing in a pUC57mini backbone the CleanCap AG-adapted T7 RNA polymerase promoter upstream of the human alpha-globin 5’ UTR (24), the 3’ UTR AES-mtRNR1, which confers increased transcript stability (25), and a segmented 100-nt poly(A) tail interrupted by a short linker (A30LA70, where L = GCAUAUGACU) (26), followed by a BspQI restriction site.

### 2.3 mRNA transcription

Plasmid DNA was linearized using the BspQI restriction enzyme (R0712S, NEB, Ipswich, MA, USA) by 16-hour digestion followed by phenol-chloroform extraction. Next, 1 µg of linearized plasmid was used as a template for *in vitro* transcription (IVT) using the HiScribe^®^ T7 Quick High Yield RNA Synthesis Kit (E2050S, NEB, Ipswich, MA, USA), incubation for 16 hours at 37°C with 4 mM CleanCap AG (TriLink Biotechnologies, San Diego, CA, USA).

*In vitro* transcribed mRNA (FABP-mRNA) was treated with DNase I (M0303, NEB, Ipswich, MA, USA) to remove the template and then precipitated by adding LiCl to a final concentration of 2.5 M, incubating for 1h at −20°C, and centrifuging for 30 min at maximum speed. Then, the pellet was washed twice with 70% ethanol. The mRNAs were resuspended in nuclease-free water and quantified using A260/A280 spectroscopy in a NanoDrop 2000 (Thermo Scientific, Waltham, MA, USA).

Subsequently, dsRNA was removed by cellulose purification (27). Briefly, 500 μg of mRNA in chromatography buffer (10 mM HEPES, 0.1 mM EDTA, 125 mM NaCl & 16% EtOH) was added to a NucleoSpin filter column (740606, Machery-Nagel) preloaded with 0.14 μg cellulose (C6288, Sigma-Aldrich). The column was shaken for 30 minutes and then centrifuged for 60 seconds at 14,000 g to collect the eluate. The eluate was precipitated with 0.1 volume of 3 M NaOAc and 1 volume of isopropanol, washed with 70% ethanol, and resuspended in nuclease-free water.

### 2.4 mRNA *in vitro* transfection

The protein production of the FABP mRNA was assessed by transfecting HEK293T cells (ATCC) cultured in DMEM (10569010, Gibco, Thermo Scientific, Waltham, MA, USA) 10% FBS (A5209502, Gibco, Thermo Scientific, Waltham, MA, USA), at 37°C with 5% CO2. Briefly, 24-well plates at 70% confluence were transfected with 10 µg of purified mRNA using 1 µL of Lipofectamine MessengerMax (LMRNA003, Invitrogen, Waltham, MA, USA) per well, according to the manufacturer’s protocol, and then incubated for 24 h. To harvest, the cells were lysed in 1x RIPA buffer (9806S, Cell Signaling Technologies, Danvers, MA, USA) with protease inhibitors (5871, Cell Signaling Technologies, Danvers, MA, USA). Cell lysates were centrifuged at maximum speed for 10 minutes, and the protein concentration in the clarified supernatant was quantified using the Pierce BCA protein assay kit (23225, Thermo Scientific, Waltham, MA, USA) and analyzed by SDS-PAGE.

### 2.5 Western blot

SDS-PAGE was used to verify protein expression in HEK-293T cells and to assess IgG presence against recombinant Fh15 (rFH15) in mouse serum. Briefly, 30 µg of lysate or the rFh15 were boiled at 95°C for 10 min in 1X SDS-PAGE Sample Loading Buffer (MB11701, NZYTech, Lisboa, Portugal) supplemented with β-mercaptoethanol. The boiled protein was separated by SDS-PAGE using precast gradient gels (MB46601, NZYTech, Lisboa, Portugal) and transferred to nitrocellulose membranes (88018, Thermo Scientific, Waltham, MA, USA). Following transfer, membranes were blocked for 60 min in 5% skimmed milk and subsequently incubated overnight at 4°C with either a 1:1000 dilution of anti-HisTag (MA121315, Invitrogen, Waltham, MA, USA) or mouse serum diluted 1:500 in 5% skimmed milk. After incubation, membranes were washed three times for 10 minutes each in 1x PBST (0.1% Tween 20). Membranes were incubated for 60 min with anti-mouse IgG-HRP secondary antibody (A9044, Sigma-Aldrich, St. Louis, MO, USA) diluted 1:10,000 in 5% skim milk in PBS. After secondary antibody incubation, membranes were washed three times for 10 minutes each in 1x PBST. Lastly, the membranes were developed by incubating for 3 min with NZY Advanced ECL (MB40201, NZYTech, Lisboa, Portugal), imaged using a ChemiDoc (Bio-Rad, Hercules, CA, USA).

### 2.6 Murine immunization

mRNA was encapsulated using a microfluidic chip through GeneScript’s custom mRNA LNP service, which employed SM102 (28) as the ionizable lipid.

**Figure 1:**
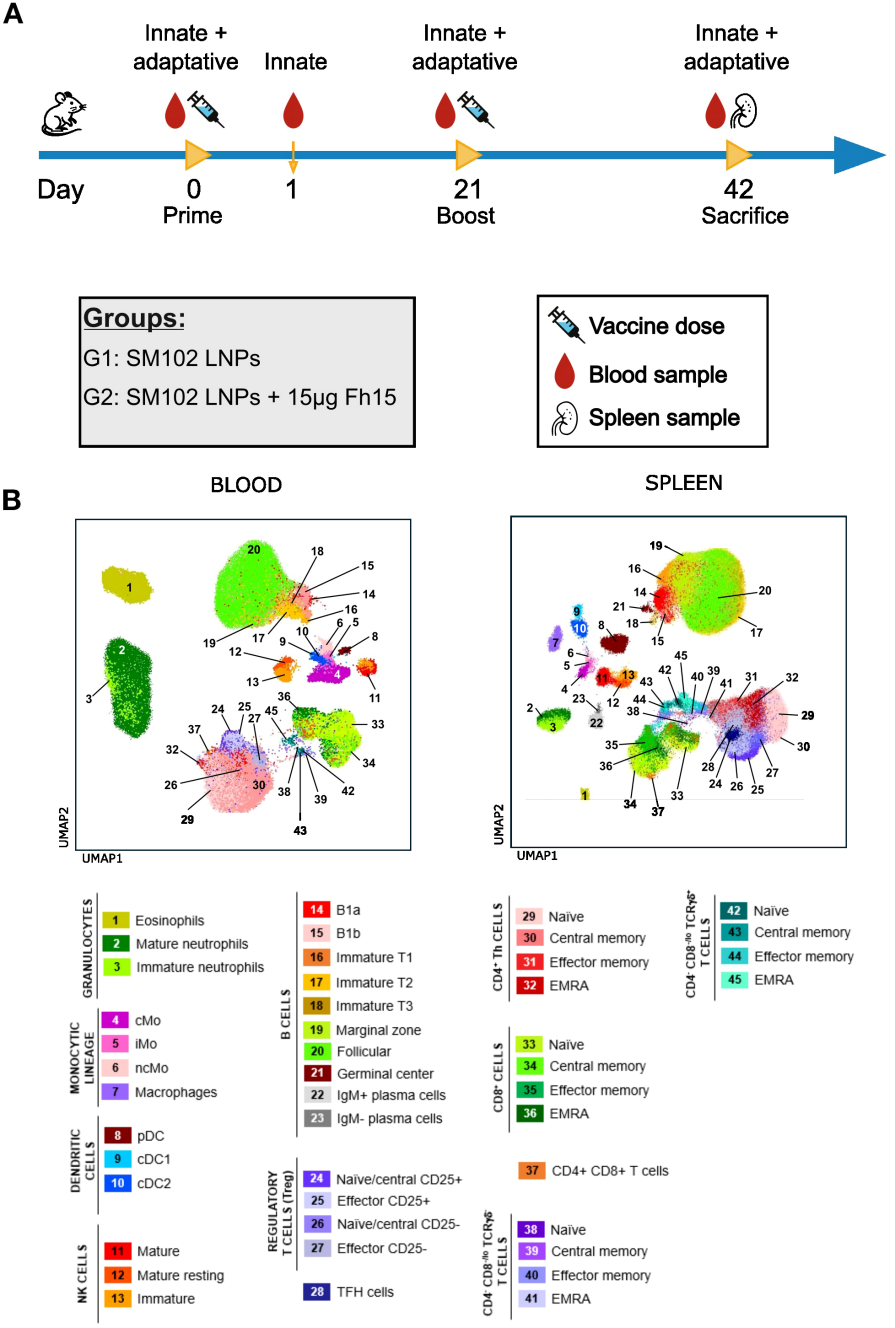
Experimental design and immune landscape of blood and spleen samples. **(A)** Schematic representation of the immunization schedule and sample collection. Mice were primed on day 0 and boosted on day 21 with SM102 LNPs (G1) or SM102 LNPs encapsulating 15 μg FABP mRNA (G2). Peripheral blood was collected at days 0, 1, 21, and 42, and spleens were collected at necropsy (day 42). **(B)** UMAP plots show the immune cell landscape in peripheral blood (left) and spleen (right) based on flow cytometry data. cMo, classical monocytes; iMo, intermediate monocytes; ncMo, non-classical monocytes; pDC, plasmacytoid dendritic cell; cDC, conventional dendritic cell; Th, T helper; TFH, T follicular helper cell; TCR, T cell receptor; NK, natural killer.

Twelve 11-week-old BALB/c female mice were randomly divided into two groups (n=6) and immunized using a prime-boost scheme (**Figure 1A**), with a 21-day interval between immunizations. Immunization was performed intramuscularly on the gastrocnemius with a 30-gauge syringe. One group received 15 μg LNP-encapsulated mRNA while the other received equivalent amounts of empty LNP with each dose. Blood samples were collected longitudinally at four time points: pre-immunization, 24 hours after the first dose, just before the second dose, and 21 days after the second dose. Spleens were collected after sacrifice at the end of the experiment and disaggregated using 0.40 μm cell strainers. Posterior analysis was performed blinded to group and mouse distribution.

### 2.7 Immunophenotypic studies

Ex vivo characterization of blood and spleen immune cells (**Figure 1B**) was performed using 50 µL of EDTA-anticoagulated, plasma-depleted blood and 2 × 10^6^ cells from spleen cell suspensions. Spleen suspensions were prepared by homogenizing spleens in PBS with a 21G syringe, followed by filtration through a 70 µm cell strainer. All samples were stained employing a stain-lyse-wash procedure. Blood samples were multiplexed (2 samples per tube) using a barcoding strategy with distinct fluorochrome-conjugated CD45 antibodies (**Supplementary Table 1**). Individual blood and spleen samples were first washed with PBS, pH 7.4 for 5 minutes at 540 x g. Samples were then pre-incubated for 30 minutes at room temperature (RT), protected from light, with the distinct barcoding antibodies (blood samples only), anti-CD3 antibody (blood and spleen samples), True-Stain Monocyte Blocker (Biolegend, San Diego, CA), TruStain FcX™ PLUS (Biolegend), and Zombie NIR viability marker (Biolegend, 1:2000 dilution). Following the pre-incubation, blood samples were washed with PBS containing 0.5% bovine serum albumin (BSA), 0.1% sodium azide, and 2 mM EDTA (pH 7.4). Samples to be multiplexed were then combined into a single tube. The remaining panel of antibodies (detailed in **Supplementary Table 1**) and Brilliant Staining Buffer Plus (BD Biosciences, San Jose, CA) were added, and samples incubated for 30 minutes at RT, protected from light, on a roller. To lyse red blood cells and fix the samples, 1X BD FACS Lysing Solution (BD Biosciences) was added and incubated for 10 minutes at RT, protected from light. Samples were subsequently washed with PBS containing 0.5% BSA, 0.1% sodium azide, and 2 mM EDTA (pH 7.4) (540 xg for 5 min), and resuspended in 400 µL of PBS before flow cytometry analysis.

### 2.8 Splenocyte culture and immunostaining

Spleens were homogenized in PBS using a 21G syringe and filtered through a 70 μm cell strainer to prepare single-cell suspensions. Splenocytes were seeded at a density of 1 x10^6^ cells/mL in 24-well plates containing 500 μL of RPMI supplemented with 2 mM L-glutamine, 1% penicillin-streptomycin, and 10% FBS for 24 hours at 37°C with 5% CO2. After this initial incubation, the cells were stimulated for 72 hours with either 10 μg/mL of recombinant Fh15 (rFh15) or 2.5 μg/mL of Phytohemagglutinin (PHA). Following incubation, cells were centrifuged (540×g for 5 min), and the supernatant was removed. Cells were then washed once with PBS containing 0.5% BSA, 0.1% sodium azide, and 2 mM EDTA (pH 7.4). Samples were multiplexed (3 samples per tube) using a barcoding strategy with distinct fluorochrome-conjugated CD45 antibodies (**Supplementary Table 1**). Samples were pre-incubated for 30 minutes at RT, protected from light, with the barcoding antibodies, True-Stain Monocyte Blocker (BioLegend), TruStain FcX™ PLUS (BioLegend), and Zombie NIR viability marker (BioLegend, 1:2000 dilution). After pre-incubation, samples were washed with PBS containing 0.5% BSA, 0.1% sodium azide, and 2 mM EDTA (pH 7.4) and combined into a single tube. Cells were then incubated with surface antibodies (**Supplementary Table 1**) for 30 minutes at RT in the dark, then fixed and permeabilized using the eBioscience™ Foxp3 Transcription Factor Staining Buffer Set (ThermoFisher Scientific, 00-5523-00) following the manufacturer’s instructions. Briefly, 1 mL of freshly prepared 1X Fixation/Permeabilization Buffer was added, and samples were incubated for 30 minutes at room temperature. After washing twice with 2 mL of 1X Permeabilization Buffer (centrifugation at 540×g for 5 min), the cells were resuspended in 100 μL of 1X Permeabilization Buffer containing intracellular antibodies (**Supplementary Table 1**) and incubated for 30 minutes at RT in the dark. Cells were then washed twice with 2 mL of 1X Permeabilization Buffer, resuspended in 300 μL of PBS, and analyzed immediately or stored at 4°C for up to 1 hour before acquisition.

### 2.9 Flow cytometry data acquisition and analysis

Data acquisition was performed on an Aurora spectral flow cytometer (Cytek, Fremont, CA) equipped with five lasers (355 nm, 405 nm, 488 nm, 561 nm, 640 nm).Daily instrument setup and quality control were performed using SpectroFlo QC beads (Cytek) according to the manufacturer’s instructions before sample measurement. To ensure accurate spectral unmixing, single-stained reference controls for each fluorochrome in the antibody panel, along with an unstained control sample, were processed identically to the multicolor-stained samples (**Supplementary Table 2**). The resulting unmixing matrix was generated using SpectroFlo software (v3.3.0; Cytek). The accuracy of the unmixing was assessed by comparing its performance with single-stained references and the full-stained sample using NxN plots.

Data analysis was conducted using Infinicyt™ software (version 2.1.0.a.000; BD Biosciences). For flow cytometric data analysis, initial gating excluded dead cells based on Zombie NIR live/dead marker expression and a parameter *vs.* time plot was employed to exclude events exhibiting unstable acquisition signal fluctuation (e.g. due to start-up/end of acquisition or clogs). Subsequently, doublets were removed by gating on forward scatter area (FSC-A) versus forward scatter height (FSC-H). Non-lysed red blood cells were excluded from the analysis using the side scatter (SSC) signal from both the blue and violet lasers. Leukocytes were defined as CD45-positive cells. Specific immune cell populations were identified based on their immunophenotypic profiles, as detailed in **Supplementary Table 3** and **Supplementary Figure 1**. T cell maturation was determined by the expression of CD27, CD44, and CD62L. Accordingly, naïve cells were defined as CD27+ CD44- CD62L+, central plus effector memory (CM+EM) as CD44+, and effector memory re-expressing CD45RA (EMRA) as CD27- CD62L- CD44-/lo). All flow cytometric data analysis was performed with Infinicyt software version 2.1.0.a (BD Biosciences, San Jose, CA, USA). Unmixed.fcs files are available at the University of Salamanca Gredos repository (http://hdl.handle.net/10366/167455).

### 2.10 Data analysis

All data were analyzed by using the R environment (29). Figures were plotted using the “ggplot2” package (30). Comparisons were made by the Wilcoxon rank sum test and exact p-values were shown. The Benjamin-Hochberg method was applied to control for the False Discovery Rate (FDR). Findings were considered statistically significant if the FDR q-value was <0.05. R files are available on Git-Hub (https://github.com/Sanchez-Montejo/Manuscript-Fh15).

## 3 Results

### 3.1 mRNA expression *of F. hepatica* FABP

**Figure 2:**
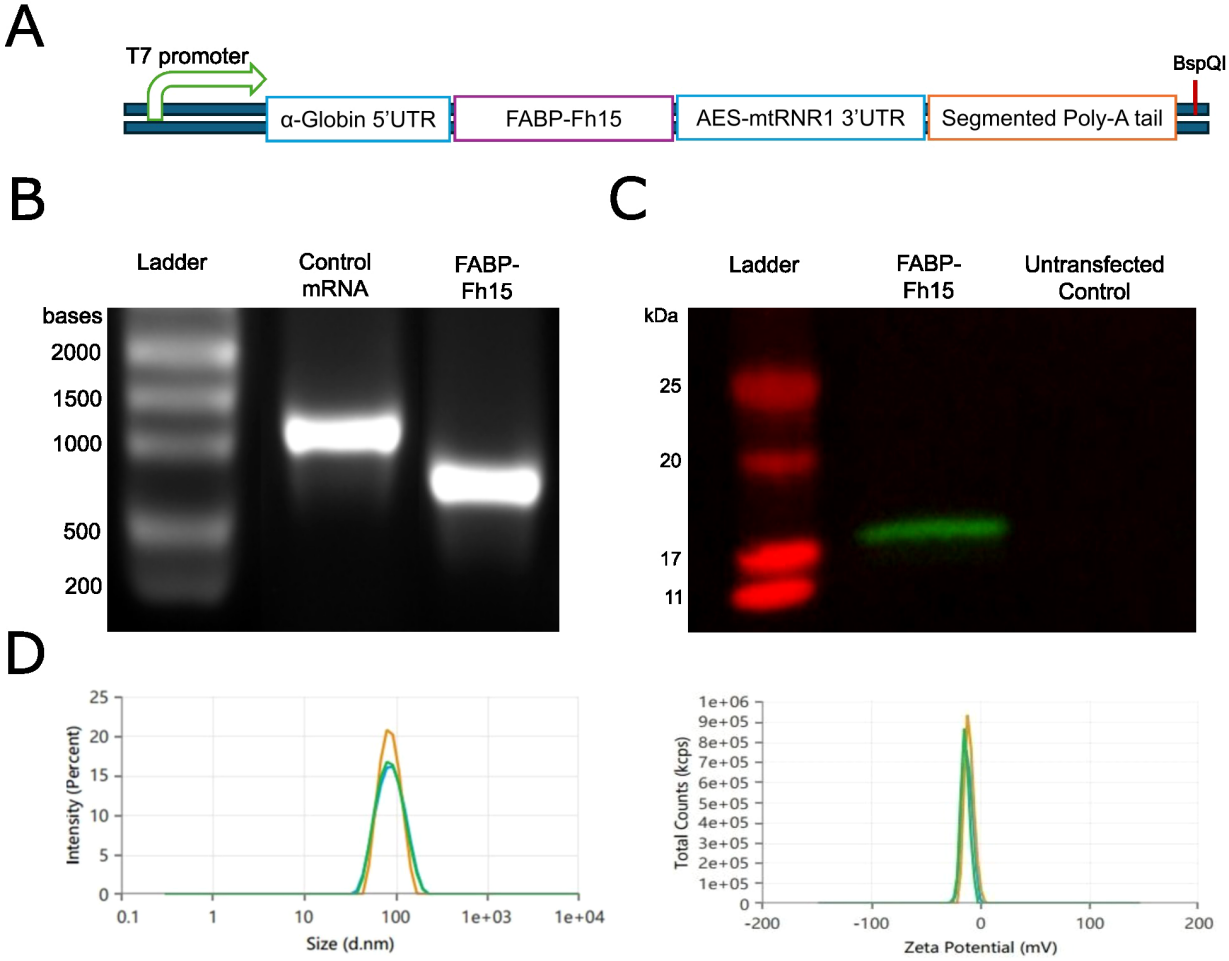
Production and characterization of FABP mRNA for immunization. **(A)** Schematic representation of the FABP mRNA expression construct indicating key elements: T7 promoter, α-Globin 5’UTR, Fh15 FABP coding sequence, AES-mtRNR1 3’UTR, and segmented poly-A tail. **(B)** Denaturing agarose gel electrophoresis analysis of purified *in vitro* transcribed (IVT) FABP and eGFP control mRNAs. **(C)** Western blot analysis of HEK293T cell lysates transfected with FABP mRNA or untransfected control. **(D)** Characterization of lipid nanoparticle (LNP) formulations encapsulating FABP mRNA, assessing particle size distribution (left) and zeta potential (right).

We optimized the sequence of *F. hepatica* FABP *Fh15* for expression in human and mouse cells, subsequently cloning it into our mRNA expression vector (**Figure 2A**). We performed *in vitro* transcription (IVT) with co-transcriptional capping and removed any possible dsRNA contamination using cellulose-packed columns. We then analyzed the transcription product using denaturing agarose gel electrophoresis to observe a noticeable band of the expected size (**Figure 2B**). Finally, we transfected the purified transcripts into HEK293T cells and analyzed protein production via western blotting (WB) with an anti-histidine antibody. The WB showed a band corresponding to the estimated size of the cloned FABP (**Figure 2C**). The capped mRNA was encapsulated into SM102 LNPs by GeneScript. The characteristics of the final particles were assessed to ensure proper mRNA encapsulation efficiency (91.65%), Z-potential (-10.2100 mV), average particle size (82.43 nm), and polydispersity index (0.08) (**Figure 2D**).

### 3.2 Mice immunized with FABP mRNA-LNP show an enhanced innate immune response

**Figure 3:**
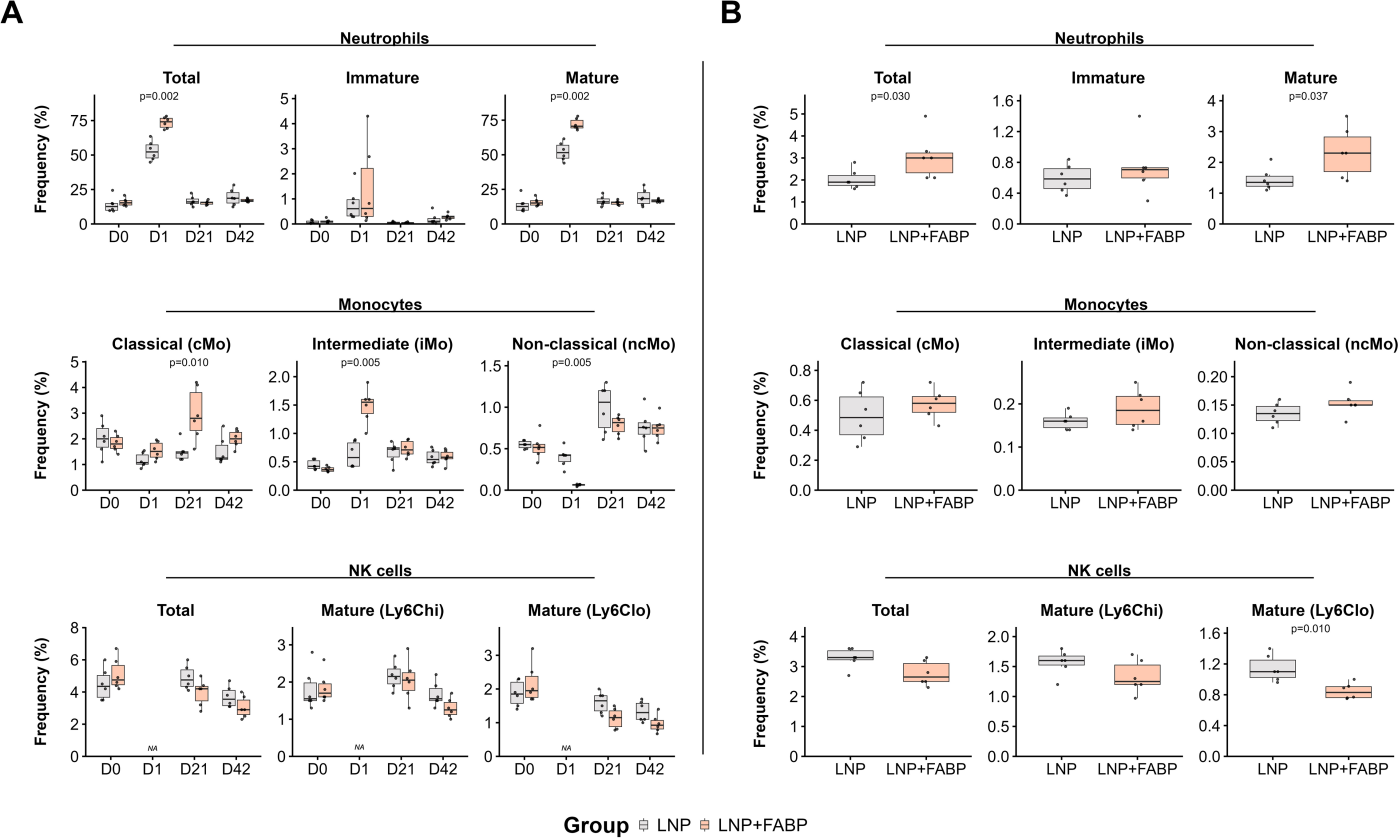
Impact of FABP mRNA-LNP vaccination on innate immune cell populations. Modulation of innate cell populations in blood and spleen in response to mRNA-LNP vaccination, as assessed by flow cytometry. **(A)** Relative frequency of neutrophil, monocytes, and NK cells, reported as percentage from total leukocytes, longitudinally evaluated in peripheral blood at different time points. **(B)** Relative frequency of neutrophils, monocytes, and NK cells, as a percentage of total leukocytes, in spleen samples after necropsy. Boxplots represent the frequency (%) of neutrophils (total, immature, mature subsets), monocytes (classical [cMo], intermediate [iMo], non-classical [ncMo] subsets), and natural killer (NK) cells (total, mature Ly6Ch^i^, mature Ly6Clo subsets). Grey indicates the empty lipid nanoparticles (LNP) control group, and salmon indicates the FABP-loaded mRNA-LNP vaccinated group (LNP+FABP). Significant differences were determined using the unpaired Wilcoxon rank-sum test; exact p-values are displayed on the graphs.

Mice immunized with either LNPs or the FABP mRNA encapsulated in LNP exhibited some degree of innate immune response (**Figure 3**). Accordingly, one day post-immunization, a significant increase in total neutrophil counts was observed in peripheral blood. This increase, reflecting a higher frequency of both mature and immature neutrophils, was observed in mice receiving either empty LNP or FABP mRNA-LNP. However, this effect was significantly more pronounced in mice immunized with the FABP mRNA vaccine, particularly for mature neutrophils (p=0.02; **Figure 3A**). A similar increase in the frequency of mature neutrophils was also observed in spleen samples from the mRNA group (**Figure 3B**). Conversely, significant sequential cellular kinetics in monocytic populations in blood were only observed in mice immunized with the LNP-encapsulated FABP mRNA. Specifically, at D1 post-immunization, non-classical monocytes (ncMo) transiently declined, suggesting their mobilization to the tissue, while a notable surge in intermediate monocytes (iMo) occurred concurrently. By 21 days post-immunization, the frequency of circulating classical monocytes (cMo) had increased significantly, likely reflecting a compensatory response and new production in the bone marrow. Interestingly, while clear kinetics were observed in blood, no significant differences between the groups were detected in the spleen samples (**Figure 3B**). Of note, no significant impact of the vaccine was observed for eosinophils, basophils, and dendritic cell populations in both blood and spleen (data not shown). Immunization with LNP-encapsulated FABP mRNA was associated with lower numbers of circulating natural killer (NK) cells, compared to empty LNP, particularly of mature NK cells with an activated/effector profile (Ly6Clo NK cells), both in blood and spleen samples. Overall, while the empty LNP induced some innate responses, mice immunized with the FABP mRNA encapsulated LNP differential innate immune response.

### 3.3 Immunization with FABP mRNA-LNP induces T-cell antigen-specific responses

**Figure 4:**
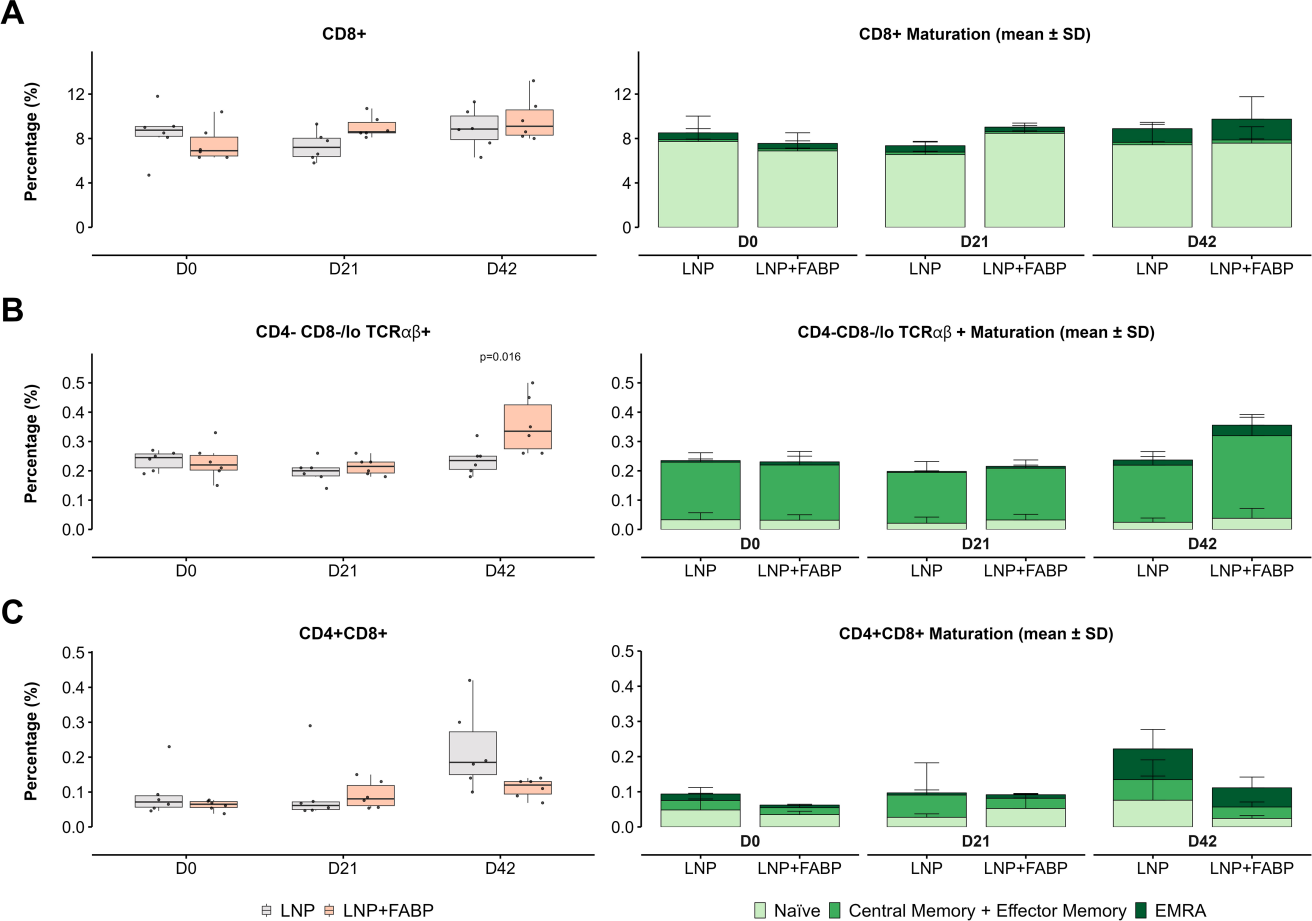
Impact of FABP mRNA-LNP immunization on blood CD8+, double negative TCRαβ+, and double positive T cell subset dynamics and maturation. Dynamic changes in CD8+, CD4−CD8−/lo TCRαβ+ and CD4+CD8+ T cell populations over time in blood in response to mRNA-LNP vaccination, assessed by flow cytometry. **(A)** CD8+ T-cells. **(B)** CD4−CD8−/lo TCRαβ+ T cells. **(C)** CD4+CD8+ T cells. Boxplots represent the relative frequency (as a percentage of total leukocytes) of the indicated populations. Grey indicates empty lipid nanoparticles (LNP) control group; salmon indicates FABP-loaded mRNA-LNP vaccinated group (LNP+FABP). Significant differences between groups and within groups over time were determined using the unpaired Wilcoxon rank-sum and Friedman tests, respectively. Exact p-values (with a false discovery rate of 5% for multiple comparison correction when applicable) are displayed on the graphs. Cumulative bar plots show the mean frequency of maturation subsets.

Overall, a significant increase in the frequency of total T cells in blood was observed over time in mice immunized with FABP mRNA-LNP (p=0.004 for D0 *vs.* D21 and p=0.02 for D0 *vs.* D42, but not for those receiving empty LNP. In line with this global T cell trend, only animals immunized with FABP mRNA-LNP displayed an increased frequency of circulating CD8+ T cells in peripheral blood (p=0.01 for D0 *vs.* D21 and D42; **Figure 4A**). Furthermore, this increase was primarily driven by effector CD8+ EMRA T-cells. Interestingly, while no significant modulation of CD4-CD8-/lo TCRγδ+ cells was observed (data not shown), CD4-CD8-/lo TCRαβ+ cells (TCRαβ+ DNT cells) exhibited a specific response to the presence of mRNA, post boosting, showing a significant increase on D42 (**Figure 4B**), with a median cell number that nearly doubled that of the control group and D0 samples. In line with CD8+ T cells, this increased frequency resulted from the expansion of central/effector memory and EMRA populations (**Figure 4B**). Conversely, CD4+ CD8+ T cells (DPT cells) displayed a trend of increased frequency in blood over time; this expansion was more pronounced in the empty LNP group at D42 (p=0.037 *vs.* FABP-loaded mRNA-LNP) (**Figure 4C**). Of note, no significant differences in the frequency of these populations were observed in the spleen (data not shown).

**Figure 5:**
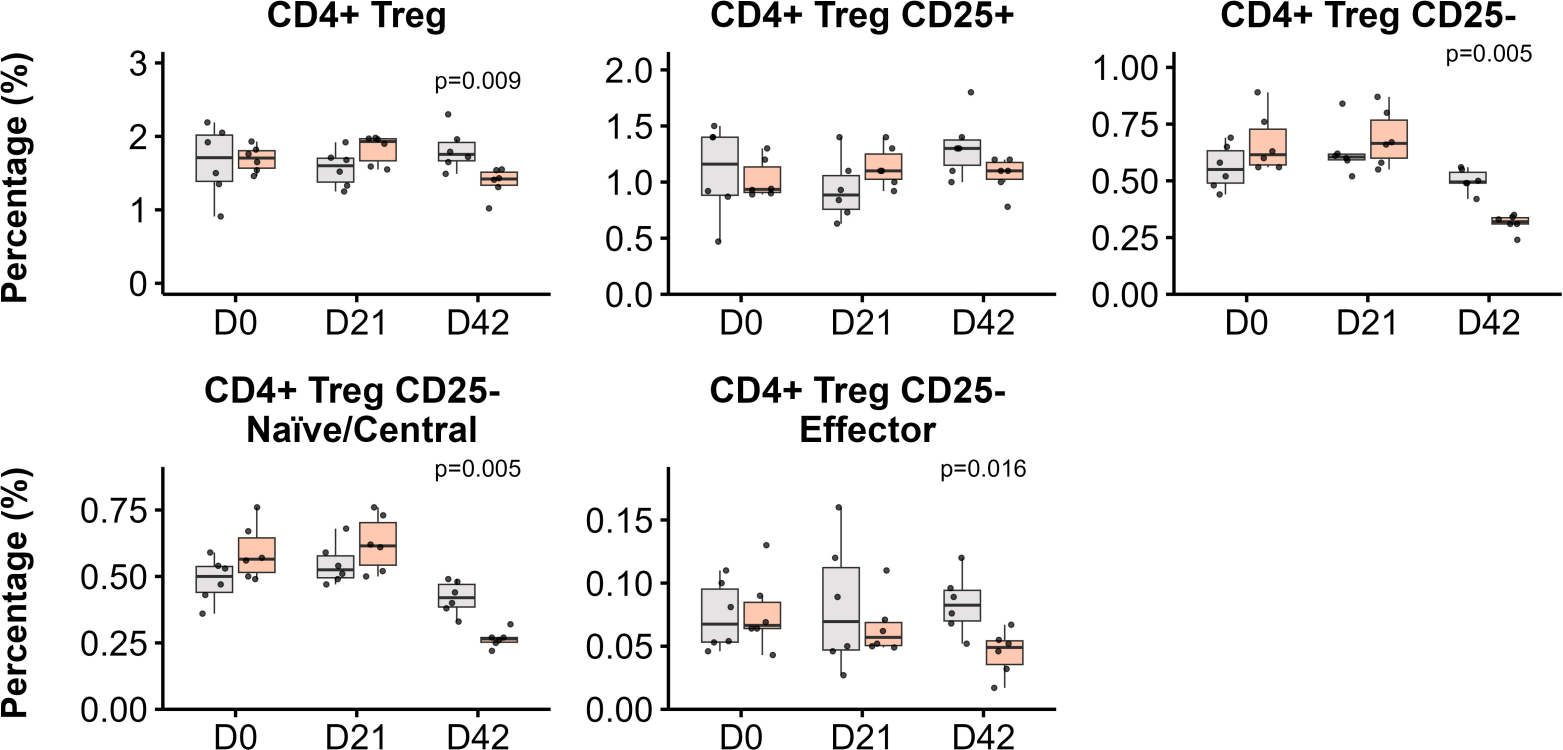
CD4+ regulatory T cell (Tregs) subpopulations in blood post-immunization. Kinetics of Treg populations in response to LNP and FABP-loaded mRNA-LNP, as assessed by flow cytometry. The relative frequencies from total leukocytes of total CD4+ Tregs, along with their CD25+ and CD25- subsets, are shown. Within the CD25- Treg population, both naïve/central and effector cell frequencies are also depicted. Boxplots represent the frequency (%) of indicated populations. Grey indicates empty lipid nanoparticles (LNP) control group; salmon indicates the FABP-loaded mRNA-LNP vaccinated group (LNP+FABP). Significant differences between groups were determined using the unpaired Wilcoxon rank-sum test; exact p-values (with a False Discovery Rate of 5% for multiple comparison correction when applicable) are displayed on the graphs.

Regulatory T cells (Tregs) also exhibited a specific modulation as a result of immunization with FABP mRNA-LNP in peripheral blood (**Figure 5**). This group showed a significant decrease in the frequency of circulating Tregs compared with the LNP-only group post-boosting (p = 0.009). This decline was primarily driven by the peripherally induced CD25- Tregs (p=0.005), with both naïve/central and effector cell populations showing diminished frequencies (**Figure 5**).

**Figure 6:**
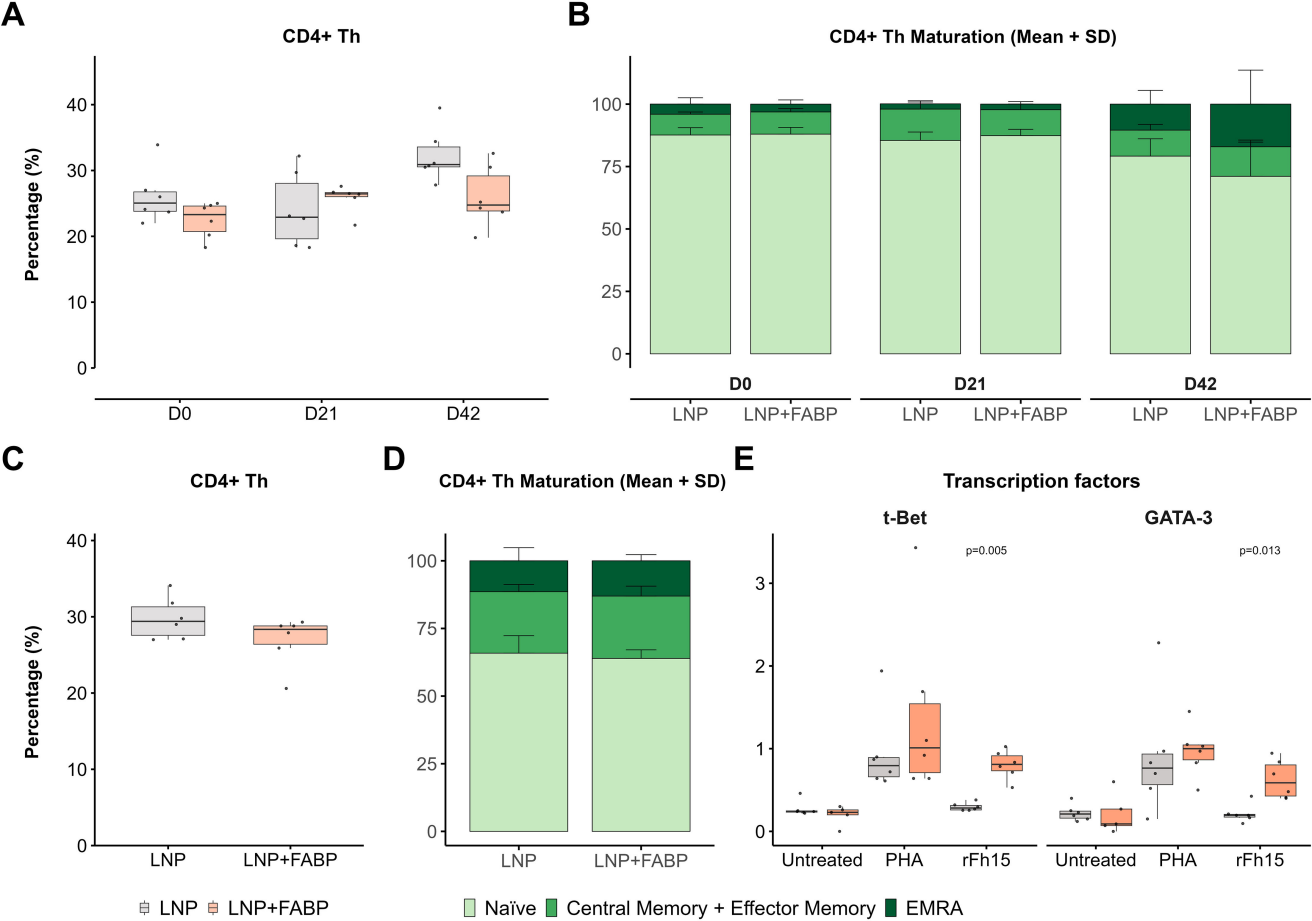
CD4+ helper T-cells (Th) after FABP mRNA-LNP immunization. Modulation of CD4+ T-helper cell populations and their antigen-specific responses following FABP mRNA-LNP immunization, as assessed by flow cytometry. **(A)** Longitudinal kinetics and maturation profile of CD4+ Th cells in peripheral blood post-immunization. Boxplots represent their frequency within total leukocytes. **(B)** Stacked bars (right) depict the relative distribution of the different maturation stages (naïve, central/effector memory, EMRA) within CD4+ Th cells. **(C)** CD4+ T-helper cell analysis in the spleen at necropsy. Boxplots show frequency within total leukocytes. **(D)** Stacked bars show maturation profile within the CD4+ Th population in spleen. **(E)** Frequency of Th1 and Th2 responses in spleen, assessed by the expression of transcription factors t-Bet and GATA-3 after *in vitro* stimulation with PHA or recombinant Fh15 protein (rFh15). Grey indicates empty lipid nanoparticles (LNP) control group; salmon indicates FABP-loaded mRNA-LNP vaccinated group (LNP+FABP). Significant differences between groups were assessed using the unpaired Wilcoxon rank-sum test; exact p-values (with a False Discovery Rate of 5% for multiple comparison correction when applicable) are displayed on the graphs.

Overall, total CD4+ T-helper (Th) cell frequency in peripheral blood showed minimal modulation after immunization, with only a slight increase observed post-boosting (median 1.3-fold for empty LNP and 1.2-fold for the FABP mRNA-LNP, compared to D0) (**Figure 6A**). However, analysis of the maturation profile of these cells revealed an increased proportion of memory and effector (CM+EM and EMRA) cells, at the expense of the naïve compartment, particularly in the FABP-loaded mRNA-LNP group (**Figure 6B**). While no significant differences in overall frequency (**Figure 6C**) and maturation profiles (**Figure 6D**) were observed between the groups in the spleen, upon stimulation with rFh15 antigen, splenocytes from mice immunized with FABP-loaded mRNA-LNP exhibited an increased frequency of antigen-specific Th1 and Th2 cells, as assessed by the expression of t-Bet and GATA-3 transcription factors (**Figure 6E**).

### 3.4 Immunization with FABP mRNA-LNP induces B-cell responses and antigen-specific antibody production

**Figure 7:**
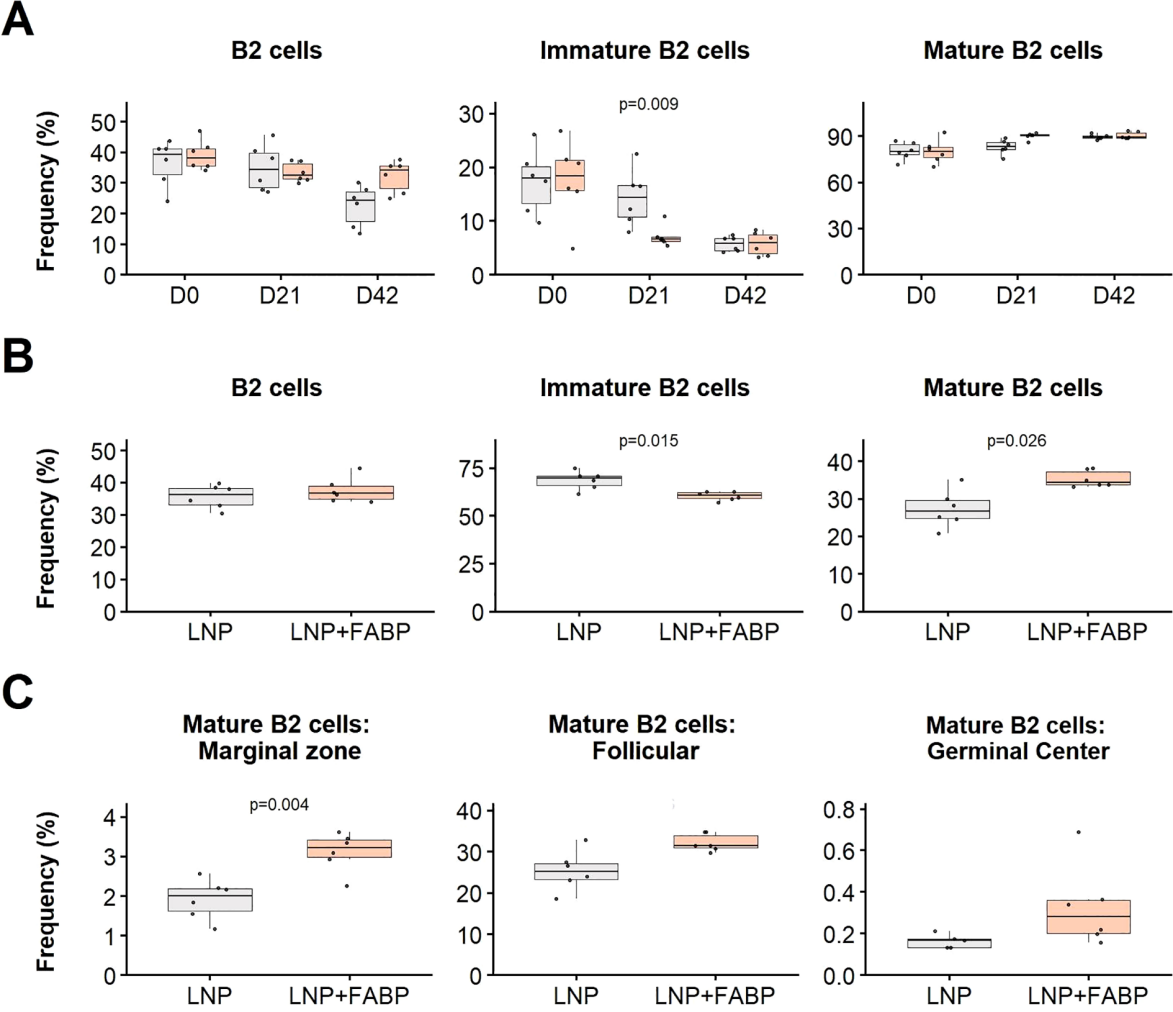
Effect of FABP mRNA-LNP immunization on B cell populations. Modulation of B2 cell subsets in blood and spleen in response to mRNA-LNP vaccination, as assessed by flow cytometry. **(A)** Relative frequency of major B2 cell subpopulations in peripheral blood at different time points post-immunization. Boxplots represent the frequency (%) of total, immature, and mature B2 cells within total leukocytes. **(B)** Relative frequency of B2 cells in the spleen at necropsy. Frequencies (%) of total, immature, and mature B2 cells are shown relative to total leukocytes. **(C)** Maturation profile of splenic mature B2 cells: marginal zone, follicular, and germinal center. Frequencies are expressed as a proportion of total B2 cells. Grey indicates empty lipid nanoparticles (LNP) control group; salmon indicates FABP-loaded mRNA-LNP vaccinated group (LNP+FABP). Significant differences between groups were determined using the unpaired Wilcoxon rank-sum test; Exact p-values (with a false discovery rate of 5% for multiple comparison correction when applicable) are displayed on the graphs.

Overall, while the FABP-loaded mRNA-LNP did not significantly affect B1 cell populations in either blood or spleen (data not shown), a significant impact was observed for different B2-cell subsets. Specifically, a substantially lower frequency of immature B cells was detected in blood and spleen in mice immunized with FABP-loaded mRNA-LNP, compared to those exposed to empty LNP (**Figures 7A, B**). This reduction in immature forms was concurrently associated with an expansion of the mature B cell compartment in both blood (D21) and spleen, suggesting the induction of B cell maturation following immunization. Interestingly, although exposure to empty LNP also induced B cell kinetics in blood, the FABP-loaded mRNA-LNP group exhibited a faster and more pronounced response. Specifically, while both groups experienced expansions of mature B2 populations, the FABP-loaded mRNA-LNP group transitioned towards mature B-cell subsets after the first dose. In contrast, the empty LNP group required a booster to achieve similar maturation effects (**Figure 7A**).

Additionally, detailed maturation profile analysis of spleen mature B2 cells revealed that immunization with the FABP mRNA led to expansion of marginal zone, follicular, and germinal center B cell subsets (**Figure 7C**), compared to empty LNP.

**Figure 8:**
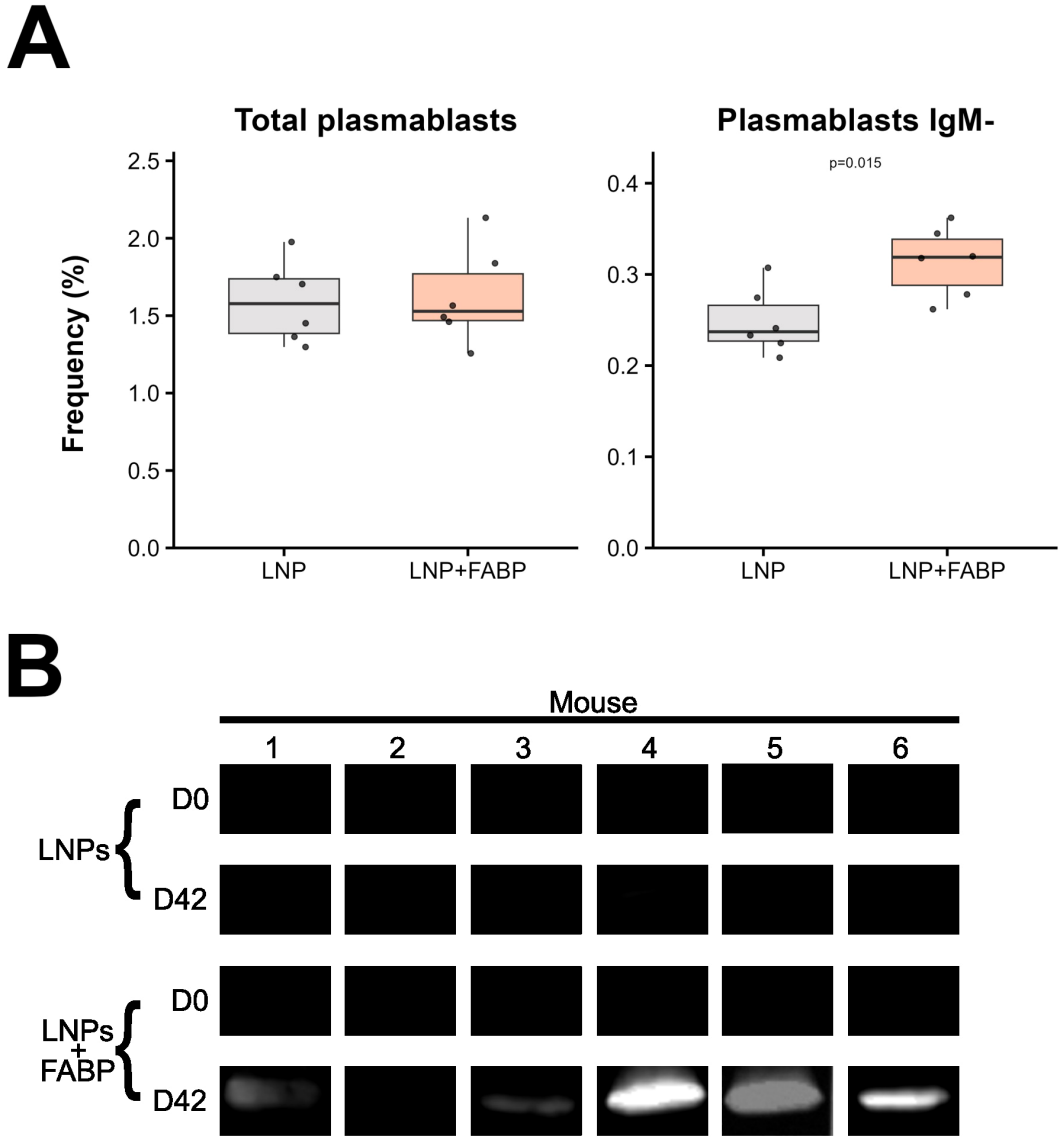
FABP mRNA-LNP immunization effect on humoral response. **(A)** Relative frequencies of total and IgM- plasma cells, as percentages of total B cells, assessed by flow cytometry in spleen samples. **(B)** Western blot analysis of serum IgG from individual mice at D0 and D42, comparing LNP and LNP+FABP groups. Grey indicates empty lipid nanoparticles (LNP) control group; salmon indicates FABP-loaded mRNA-LNP vaccinated group (LNP+FABP). Significant differences were determined using the unpaired Wilcoxon rank-sum test; exact p-values (with a false discovery rate of 5% for multiple comparison correction when applicable) are displayed on the graphs.

Finally, the humoral response generated by the immunization was evaluated. Interestingly, despite no significant differences in plasma cell frequency being observed in the spleen post-boosting (**Figure 8A**), the frequency of class-switched (IgM^-^) plasma cells was significantly higher in mice immunized with the FABP-loaded mRNA-LNP. This was further corroborated by the detection of IgG antibodies recognizing the recombinant protein rFh15 in plasma by Western Blot (**Figure 8B**), which was observed exclusively in the mRNA-vaccinated group.

## 4 Discussion

The primary aim of this study was to develop and characterize an mRNA-based vaccine using the FABP protein (Fh15) of *Fasciola hepatica*, a primary causative agent of fasciolosis. In this study, we optimized the sequence of the FABP Fh15 and cloned it into an mRNA vector, which was then expressed *in vitro* using the HEK293T cell line and encapsulated in LNPs for mouse immunization.

Mice immunized with FABP mRNA-LNP exhibited rapid innate immune activation, with early mobilization and maturation of neutrophils. As expected during acute inflammation, immature neutrophils increased in circulation, compensating for tissue migration (“left shift”) (31). This is consistent with local inflammation induced by mRNA LNP-based vaccines, where local inflammation is induced at the injection site due to the presence of the LNPs (32) and the presence of unmodified mRNA (33). In contrast, monocyte responses were specific to FABP mRNA-LNP. At 24 h post-immunization, patrolling ncMo were depleted, suggesting migration to inflamed tissues (34). This decrease in ncMo at D1 was associated with a nearly 3-fold increase in circulating iMo. Notably, while mouse iMo are less characterized, the intermediate CD14+/CD16+ monocyte subset is characterized by high surface expression of antigen-presenting and co-stimulatory molecules, including HLA-DR, CD80, and CD86 (35). Our observation of increased intermediate monocytes (with presumably higher MHC-II, CD80, and CD86 expression) in the FABP-immunized group suggests ongoing antigen immune activation, consistent with prior reports that intermediate monocytes upregulate these markers during infection or immunization (36). Furthermore, the rise in circulating iMo aligns with an inflammatory response, as has been documented in various human disease conditions, including malaria (37). Interestingly, despite clear monocyte kinetics being observed in blood specifically in response to FABP-loaded mRNA-LNP immunization, no significant differences were observed in the spleen for these populations. The spleen is a critical reservoir for monocytes, providing a large and rapidly deployable pool that complements the continuous surveillance and initial response of blood monocytes. This splenic reserve is crucial for combating larger infections and managing significant inflammatory events (38). Therefore, the lack of kinetics observed in spleen monocytic populations suggests that the local inflammation, induced by the vaccine inoculation, was limited, not requiring the deployment of the spleen reservoir. Overall, the additive effect on neutrophils and specific modulation patterns of monocytes suggest that, upon inoculation, the FABP is rapidly produced (within 24 hours) and is immunogenic, capable of triggering an inflammatory response that remains immunologically controlled, thereby avoiding extreme inflammation, a significant safety concern for vaccine development. However, the lack of a mock mRNA in the control group difficulties the discrimination of the specific effect of the FABP versus an irrelevant mRNA. Additionally, we observed a decrease in circulating mature NK cells, particularly those with activated/effector functions (Ly6Clo), after each dose. This reduction would be consistent with the recruitment of these cells to lymphoid organs (e.g., draining lymph nodes) upon activation (39), although this should be properly confirmed by analyzing in a new vaccination experiment. Similar findings have been reported with other mRNA vaccines, where activated NK cells and other lymphocytes traffic to lymph nodes as part of the vaccine response, as it induces the recruitment of IL6+ dendritic cells and promotes the B cell response (40). In this study, we did not find significant differences in the relative frequency of dendritic cells in the peripheral blood or spleen, likely due to their accumulation in draining lymph nodes or the injection site (32). This should be specifically addressed by studying lymph node populations in future studies.

Evaluation of cellular immune responses following immunization with the FABP mRNA-LNP formulation showed a significant increase in TCRαβ+ DN T-cell populations, especially within the effector (EMRA) subset, known to promote Th1, Th2, and Th17 responses and to be key in immunity against parasites like *Leishmania* spp. (41, 42). In contrast, while double-positive CD4+CD8+ T cells were significantly increased at D42 in the group immunized with empty LNP, a lower frequency of these cells was observed for the mice exposed to the complete vaccine. Since there was no overall decrease in circulating DP T cells in the complete vaccine group, but rather a shift toward a memory/effector phenotype, this lower frequency likely indicates their potential migration to tissues. DP T cells were antigen-reactive lymphocytes in the self-cure of *Schistosoma* infections in rhesus macaques (43). This suggests that they could also produce antigen recognition against *Fasciola*, contributing to protective immunity.

LNPs serve as the primary adjuvants for T helper cell stimulation in mRNA immunization (44). In line with this, both study groups exhibited a minor relative frequency increase over time, more pronounced in the control group. Interestingly, despite minor overall differences between groups, which were modest by day 42, significantly different maturation profiles were observed compared to day 0. Both groups showed higher proportions of memory and effector cells, with more pronounced changes in the mRNA group. This pattern suggests that after the booster dose, antigen-specific CD4+ T cells may have migrated to peripheral sites to aid B cell responses. Although no significant differences in total CD4+ T cell frequencies were observed in the spleen, a significantly increased frequency of antigen-specific Th1 and Th2 cells was detected exclusively in the group immunized with FABP-loaded mRNA-LNP. The induction of both responses might help fight the parasite, as *Fasciola* represses Th1 responses and induces Th2 responses (45). While functional assays, including intracellular cytokine staining/ELIspot and cytotoxicity assays, would provide a definitive measure of immediate T-cell effector function, their inclusion in this study was feasible due to severe limitations in splenocyte availability and the imperative to adhere to the 3R principle of Reduction in animal use. Our decision prioritized T-bet and GATA-3 staining at the 72h time point, which provides a robust measure of vaccine-induced Th1/Th2 lineage commitment and clonal expansion, the central aim of our T-cell analysis.

Finally, we demonstrated that our FABP mRNA induced a humoral response, specifically in the mice that received the mRNA. While both experimental groups showed a significant decrease in immature B2 cells and an increase in mature B2 cells in peripheral blood post-boosting, the timing of these responses differed notably between groups. Mice receiving the mRNA showed a rapid B2 cell kinetic immediately after the first dose, whereas the control group reached similar levels only after the second dose. The lack of significant differences between groups after the second dose, associated with the comparable levels of total plasma cells in the spleen, suggests that the lipid nanoparticle vehicle alone may also trigger some B cell immune response, potentially T-cell independent (46). In line with this, expansion of mature B2 cells in the spleen in mice immunized with FABP-loaded mRNA-LNP reflected the significant increase in the relative frequency of follicular, marginal zone, and germinal center B2 cells. This occurred at the expense of immature cells, ultimately indicating vaccine-induced B cell maturation. Interestingly, despite no significant differences being observed in the frequency of plasma cells in the spleen between the two groups, mice exposed to the complete vaccine displayed significantly higher frequency of class-switched, IgG-producing plasma cells. This IgG production aligned with the positive detection of the target antigen by western blot, supporting the specificity of the humoral response generated by our mRNA and suggesting that the two-dose FABP-loaded mRNA-LNP vaccination scheme successfully elicited robust antigen-specific primary and class-switched secondary immune responses. These results are similar to those of other mRNA vaccines, which enhance antigen-specific responses and stimulate the activation of germinal centers, ultimately leading to the production of neutralizing antibodies against viruses (47). Notably, the mRNA immunization of *Necator americanus* proteins produced similar maturation of T cells and antigen-specific antibody production (22).

Despite the extensive scientific efforts over the past decades to develop an effective vaccine against *Fasciola hepatica*, the results have been inconsistent and have not led to a satisfactory vaccine (48). Our results demonstrate that the evaluated mRNA can effectively produce a *Fasciola hepatica* protein *in vivo* and induce an immune response that produces both humoral and cellular responses. Further experiments are needed to test this mRNA vaccine against parasite infection, testing whether the effect of the described immune response provides protection against the parasite. However, these experiments have established a foundation for developing an effective mRNA vaccine against *Fasciola hepatica.*

## Data Availability

The original contributions presented in the study are included in the article/**Supplementary Material** the dataset and data management are available at the GREDOS repository at the University of Salamanca (DOI:10.71636/zmp6-m645) in http://hdl.handle.net/10366/167455. Data management is available on GitHub (https://github.com/Sanchez-Montejo/Manuscript-Fh15). Further inquiries can be directed to the corresponding author/s.
