# Supplementary material for: Harnessing mRNA technology against Fasciola hepatica: Immunological insights from a fatty acid binding protein vaccine
Source: Front Immunol. 2025 Nov 25;16:1693674. doi: 10.3389/fimmu.2025.1693674 (PMC12687294; doi:10.3389/fimmu.2025.1693674)
Supplement: Supplementary file 2 [file Table2.docx]

**Supplementary Table 2**. Single-stained reference controls employed for spectral unmixing.

| **Antibody** | **Type of particle** | **Positive reference population** |
| --- | --- | --- |
| CD3 PE Fire700 | Beads | Beads |
| CD4+ IgD BV605 | Cells (pre-wash) | Lymphocytes |
| CD5 BV750 | Beads | Beads |
| CD8a + IgM BUV395 | Cells (pre-wash) | Lymphocytes |
| CD11b BV421 | Beads | Beads |
| CD11c BUV615 | Cells | Monocytes |
| CD19 + TCRβ RB545 | Cells | Lymphocytes |
| CD21/CD35 APC | Cells | Lymphocytes |
| CD23 BUV737 | Cells | Lymphocytes |
| CD25 BV480 | Beads | Beads |
| CD27 BB700 | Cells | Lymphocytes |
| CD43 BV510 | Cells | Monocytes |
| CD44 APC Vio770 | Cells | Lymphocytes |
| CD45 BUV496 | Cells | Lymphocytes |
| CD45 APC | Cells | Lymphocytes |
| CD45.2 PerCP | Cells | Lymphocytes |
| CD45R (B220) VioBlue | Cells | Lymphocytes |
| CD62L BUV805 | Cells | Lymphocytes |
| CD64 PE | Beads | Beads |
| CD69 BUV661 | Beads | Beads |
| CD93 RY586 | Cells | Lymphocytes |
| CD95 RB744 | Beads | Beads |
| CD103 PE Fire640 | Cells | Lymphocytes |
| CD127 RB780 | Cells | Lymphocytes |
| CD138 PE Cy5 | Beads | Beads |
| CD185 PE CF594 | Beads | Beads |
| CD279 (PD1) APC Fire810 | Beads | Beads |
| CD304 BUV563 | Cells | Lymphocytes |
| CD335 (NKp46) BV711 | Beads | Beads |
| F4/80 SparkNIR685 | Beads | Beads |
| GL7 RB705 | Beads | Beads |
| I-A/I-E (MHC-II) BV650 | Cells | Lymphocytes |
| Ly6C PE Fire810 | Cells | Monocytes |
| Ly6G Ly6G | Cells | Neutrophils |
| NK1.1 BV570 | Beads | Beads |
| Siglec F AF647 | Beads | Beads |
| t-Bet BV786 | Beads | Beads |
| GATA-3 PE Cy7 | Beads | Beads |
| Zombie NIR | Cells* | Dead cells |

* Induce cell death prior to staining by incubating the cells at 60ºC for 10min prior to staining

Beads employed for spectral unmixing: UltraComp eBeads^TM^ (Thermo Fisher Scientific, cat. nr. 01-2222-42)

*Abbreviations:* AF, Alexa fluor; APC, allophycocyanin; BB; Brilliant Blue; BUV, Brilliant Ultraviolet; BV, Brilliant Violet; CF, Cyanin-based Fluorescent dye; Cy7, Cyanin7; N/A, not applicable; NIR, near-infrared; PE, Phycoerythrin; PerCP Cy5.5, Peridinin-chlorophyll-protein-cyanin 5.5; RB, RealBlue; RY, RealYellow; R, Red;
