# Supplementary material for: Harnessing mRNA technology against Fasciola hepatica: Immunological insights from a fatty acid binding protein vaccine
Source: Front Immunol. 2025 Nov 25;16:1693674. doi: 10.3389/fimmu.2025.1693674 (PMC12687294; doi:10.3389/fimmu.2025.1693674)
Supplement: Supplementary file 3 [file Table3.docx]

**Supplementary Table 3.** Immunophenotypic profiles employed for population identification in blood and spleen.

| **Leukocyte population** | **Immunophenotype** |
| --- | --- |
| *Eosinophils* | SSC^hi^, Siglec F^+^, CD11c^-/lo^, CD3^-^, CD19^-^, CD335^-^, F4/80^-^, Ly6G^-^ |
| *Total neutrophils* | SSC^hi^, Ly6G^lo/+^, CD11b^lo/+^, CD3^-^, CD11c^-^, CD19^-^, CD335^-^, F4/80^-^, Siglec F^-^ |
| *Immature neutrophils* | SSC^hi^, Ly6G^lo^, CD11b^lo/+^, CD3^-^, CD11c^-^, CD19^-^, CD335^-^, F4/80^-^, Siglec F^-^ |
| *Mature neutrophils* | SSC^hi^, Ly6G^+^, CD11b^/+^, CD3^-^, CD11c^-^, CD19^-^, CD335^-^, F4/80^-^, Siglec F^-^ |
| *Monocytes* | CD43^-/+^, Ly6C^-/+^, I-A/I-E^-/lo^, CD3^-^, CD19^-^, CD335^-^, F4/80^-^, Ly6G^-^, Siglec F^-^ |
| *Classical monocytes* | CD43^-^, Ly6C^+^, I-A/I-E^-/lo^, CD3^-^, CD19^-^, CD335^-^, F4/80^-^, Ly6G^-^, Siglec F^-^ |
| *Intermediate monocytes* | CD43^+^, Ly6C^+^, I-A/I-E^-/lo^, CD3^-^, CD19^-^, CD335^-^, F4/80^-^, Ly6G^-^, Siglec F^-^ |
| *Non-classical monocytes* | CD43^+^, Ly6C^-^, I-A/I-E^-/lo^, CD3^-^, CD19^-^, CD335^-^, F4/80^-^, Ly6G^-^, Siglec F^-^ |
| *Macrophages* | SSC^hi^ ,CD11b^+^, CD64^+^, F4/80^lo/++^, I-A/I-El^o/+^, CD3^-^, CD19^-^, CD335^-^, Ly6G^-^, Siglec F^-^, |
| *Dendritic cells (DC)* | B220^-/+^, CD11b^-/+^ CD11c^-/+^, CD8a^-/+^, CD103^-/+^, I-A/I-E^+/++^, CD3^-^, CD19^-^, CD335^-^, F4/80^-^, Ly6G^-^, Siglec F^-^ |
| *Plasmacytoid DC* | B220^+^, CD11b^-^ CD11c^-/+^, CD8a^-^, CD103^-^, I-A/I-E^+^, CD3^-^, CD19^-^, CD335^-^, F4/80^-^, Ly6G^-^, Siglec F^-^ |
| *Myeloid DC* | B220^-^, CD11b^-/+^ CD11c^+^, CD8a^-/+^, CD103^-/+^, I-A/I-E^++^, CD3^-^, CD19^-^, CD335^-^, F4/80^-^, Ly6G^-^, Siglec F^-^ |
| *cDC2* | B220^-^, CD11b^+^ CD11c^+^, CD8a^-^, CD103^-^, I-A/I-E^++^, CD3^-^, CD19^-^, CD335^-^, F4/80^-^, Ly6G^-^, Siglec F^-^ |
| *cDC1* | B220^-^, CD11b^-/lo^ CD11c^+^, (CD8a and/or CD103)^+^, I-A/I-E^++^, CD3^-^, CD19^-^, CD335^-^, F4/80^-^, Ly6G^-^, Siglec F^-^ |
| *Total lymphocytes* | SSC^lo/int^, CD45^hi^ (B220^-/+^ and/or CD3^-/+^ and/or CD19^-/+^ and/or CD335^-/+^) |
| *NK cells* | SSC^lo^, CD45^hi^, CD335+, B220^-^, CD3^-^, CD19^-^ |
| *Immature NK cells* | SSC^lo^, CD45^hi^, CD335+, CD11b^lo^, CD27^+^, B220^-^, CD3^-^, CD19^-^ |
| *Mature NK cells* | SSC^lo^, CD45^hi^, CD335+, CD11b^+^, CD27^lo^, B220^-^, CD3^-^, CD19^-^ |
| *Mature resting NK cells* | SSC^lo^, CD45^hi^, CD335+, CD11b^+^, CD27^lo^, Ly6C^+^, B220^-^, CD3^-^, CD19^-^ |
| *Mature non-resting NK cells* | SSC^lo^, CD45^hi^, CD335+, CD11b^+^, CD27^lo^, Ly6C^-/lo^, B220^-^, CD3^-^, CD19^-^ |
| *T cells* | SSC^lo^, CD45^hi^, CD3^+^, TCRβ^-/+^, CD19^-^, CD335^-^ |
| *CD4^+^ CD8^-^ T cells* | SSC^lo^, CD45^hi^, CD3^+^, CD4^+^, CD8^-^, TCRβ^+^, CD19^-^, CD335^-^ |
| *TFH* | SSC^lo^, CD45^hi^, CD3^+^, CD4^+^, CD8^-^, CD185^+^, CD279^+^, TCRβ^+^, CD19^-^, CD335^-^ |
| *Treg* | SSC^lo^, CD45^hi^, CD3^+^, CD4^+^, CD8^-^, CD127^lo^, (CD25^+^ and/or CD304^+^), TCRβ^+^, CD19^-^, CD335^-^ |
| *CD25^+^ Treg* | SSC^lo^, CD45^hi^, CD3^+^, CD4^+^, CD8^-^, CD25^+^, CD127^lo^, CD304^-/+^, TCRβ^+^, CD19^-^, CD335^-^ |
| *CD25^-^ Treg* | SSC^lo^, CD45^hi^, CD3^+^, CD4^+^, CD8^-^, CD25^-^, CD127^lo^, CD304^+^, TCRβ^+^, CD19^-^, CD335^-^ |
| *CD4+ Th cells* | SSC^lo^, CD45^hi^, CD3^+^, CD4^+^, CD8^-^, CD127^+^, TCRβ^+^, CD19^-^, CD335^-^ |
| *CD4^-^ CD8^+^ T cells* | SSC^lo^, CD45^hi^, CD3^+^, CD4^-^, CD8^+^, TCRβ^+^, CD19^-^, CD335^-^ |
| *CD4^+^ CD8^+^ T cells* | SSC^lo^, CD45^hi^, CD3^+^, CD4^+^, CD8^+^, TCRβ^+^, CD19^-^, CD335^-^ |
| *CD4^-^ CD8^-/lo^ TCRαβ+ T cells* | SSC^lo^, CD45^hi^, CD3^+^, CD4^-^, CD8^-/lo^, TCRβ^+^, CD19^-^, CD335^-^ |
| *CD4^-^ CD8^-/lo^ TCRγδ+ T cells* | SSC^lo^, CD45^hi^, CD3^+^, CD4^-^, CD8^-/lo^, TCRβ^-^, CD19^-^, CD335^-^ |
| *B cells* | SSC^lo/int^, CD45^hi^, B220^-/+^, CD19^+^, CD3^-^, CD335^-^ |
| *B1 B cells* | SSC^lo^, CD45^hi^, B220^-/lo^, CD19^+^, CD3^-^, CD335^-^ |
| *B1a B cells* | SSC^lo^, CD45^hi^, B220^-/lo^, CD5^+^, CD19^+^, CD3^-^, CD335^-^ |
| *B1b B cells* | SSC^lo^, CD45^hi^, B220^+^, CD5^-^, CD19^+^, CD3^-^, CD335^-^ |
| *B2 B cells* | SSC^lo^, CD45^hi^, B220^+^, CD19^+^, CD3^-^, CD335^-^ |
| *Immature B2 cells* | SSC^lo^, CD45^hi^, B220^+^, CD19^+^, CD93+, IgM^-/+^, CD23^-/+^, CD3^-^, CD335^-^ |
| *Immature T1 cells* | SSC^lo^, CD45^hi^, B220^+^, CD19^+^, CD93+, IgM^+^, CD23^-^ |
| *Immature T2 cells* | SSC^lo^, CD45^hi^, B220^+^, CD19^+^, CD93+, IgM^+^, CD23^+^ |
| *Immature T3 cells* | SSC^lo^, CD45^hi^, B220^+^, CD19^+^, CD93+, IgM^-/lo^, CD23^+^ |
| *Mature MZ B2 cells* | SSC^lo^, CD45^hi^, B220^+^, CD19^+^, CD21/CD35^hi^, CD23^lo^, CD43^-^, CD93^-^, IgD^lo^, IgM^hi^, CD3^-^, CD335^-^ |
| *Mature Follicular B2 cells* | SSC^lo^, CD45^hi^, B220^+^, CD19^+^, CD21/CD35^lo^, CD23^hi^, CD43^+^, CD93^-^, IgD^hi^, IgM^lo^, CD3^-^, CD335^-^ |
| *Mature GC B2 cells* | SSC^lo^, CD45^hi^, B220^+^, CD19^+^, CD93^-^, CD95^+^, GL7^+^, CD3^-^, CD335^-^ |
| *Plasmablasts/plasma cells* | SSC^lo/int^, CD45^+^, B220^-/lo^, CD19^+^, CD138^+^, CD3^-^, CD335^-^ |

***Abbreviations:*** *cDC, Conventional dendritic cells; hi, high; int, intermediate; GC, germinal center; lo, low; MZ, marginal zone; SSC, side scatter; TFH, T follicular helper cells; Th, T helper; Treg, regulatory T cells.*
