# Supplementary material for: Harnessing mRNA technology against Fasciola hepatica: Immunological insights from a fatty acid binding protein vaccine
Source: Front Immunol. 2025 Nov 25;16:1693674. doi: 10.3389/fimmu.2025.1693674 (PMC12687294; doi:10.3389/fimmu.2025.1693674)
Supplement: Supplementary file 4 [file DataSheet1.docx]

**
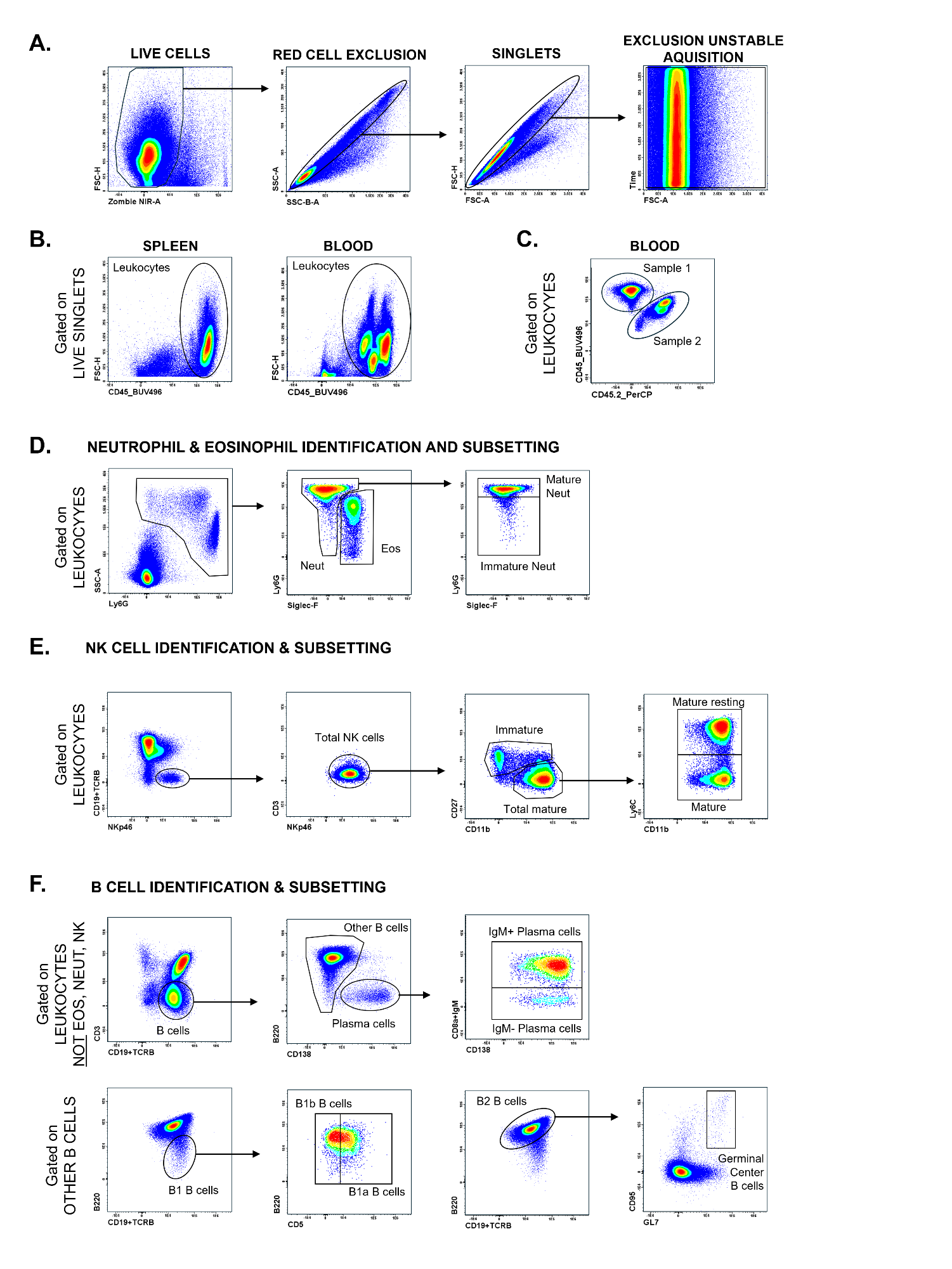
**

**
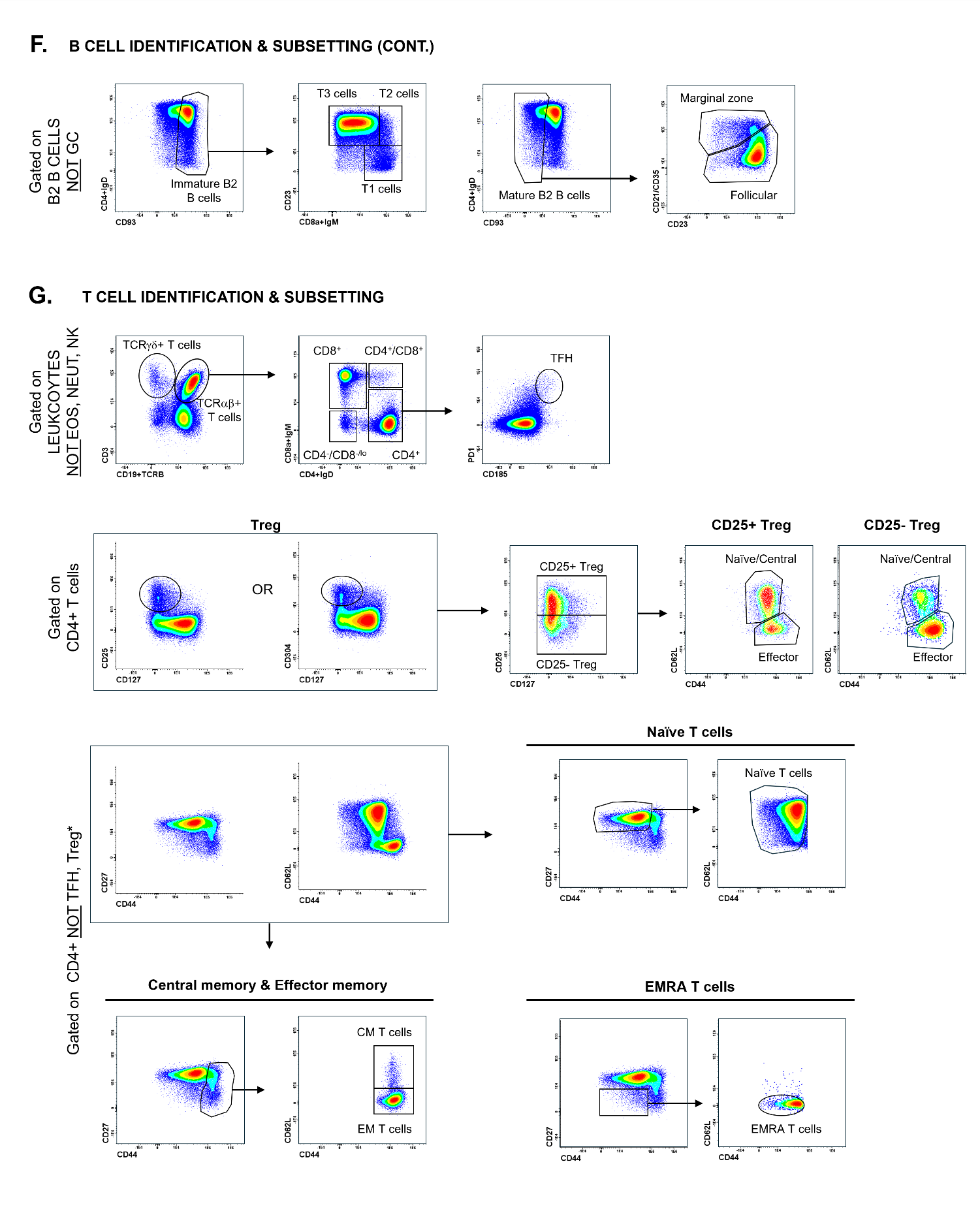
**

**
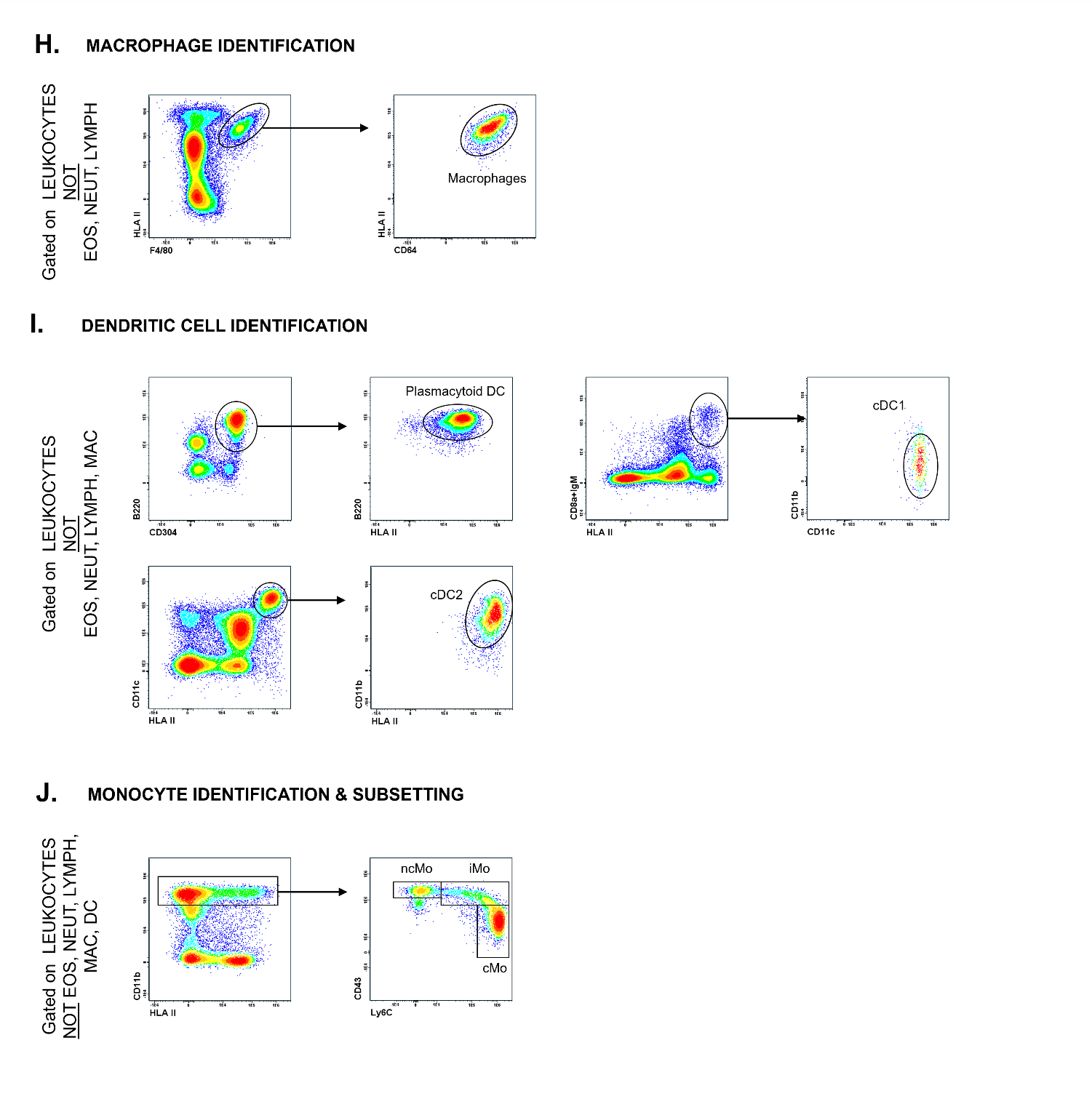
**

**Supplementary Figure 1. Gating strategy employed for identification of immune cell populations in blood and spleen**. Panel A shows the clean up and quality control steps, including selection of live singlets, and exclusion of non-lysed red cells and of unstable acquisition. Panel B depicts the identification of leukocytes in spleen and blood, while Panel C shows the demultiplexing of the barcoding performed in blood samples. Identification and subsetting of eosinophils and neutrophils, NK cells and B cells are indicated in Panels D, E and F, respectively. Panel G reports the strategy for identification of the major T cell subsets, including regulatory T cells, T follicular helper cells, as well as a representative example of the strategy for evaluation of T cell maturation stages. Panels H, I and J exhibit the gating strategy for identification of macrophage, dendritic cell and monocytic populations, respectively.

*Eos, eosinophils; Neut, neutrophils; NK, natural killer; TFH, T follicular helper cells; Treg, regulatory T cells; GC, germinal center B cells; EMRA, terminally differentiated effector memory cells re-expressing CD45RA; DC, dendritic cell; cDC1, type 1 conventional dendritic cell; cDC2, type 2 conventional dendritic cell; Lymph, lymphocytes; Mac, macrophages; cMo, classical monocytes; iMo, intermediate monocytes; ncMo, non-classical monocytes.*
